# Hospital Worthies

**Published:** 1887-07-30

**Authors:** 


### Hospital Worthies.

By a Reader of Biographies.

### JOHN RADCLIFFE, M.D.

Wit and Physic.

It would be difficult to say whether it was for his physic or his wit that this worthy most excelled, but, while the former frequently cured, it must be frankly confessed that the latter generally galled. Dr. Radcliffe cared for kings and queens no more than he did for his humbler patients, and if they did not relish his caustic jibes, they might just do "the other thing."

Boyhood.

John Radcliffe first saw the light of day at Wakefield, 7th February 1650. His father possessed a moderate estate, and though himself "more addicted to the improvement of his paternal acres than the cultivation of letters," he listened to the advice of some of his friends and neighbours, and, after John had attended for a time the local grammar school, sent him, at the early age of fifteen, to Oxford.

A Strange Library.

He had not been long at Oxford before he determined to make physic his study. But, in the practice of the healing art, he resolved he would not be hampered by the musty tomes of the ancients; and even with regard to contemporary writers he was exceedingly sparing in the acquisition of books. These were so few in number as on one occasion to elicit an expression of surprise from the Master of Trinity, who happened to visit his "college." "Sir," said the young student to Dr. Bathurst, "there is Radcliffe's library," pointing to some glass vials, a herbal, and a skeleton, which lay heaped in a corner.

Onward.

If Radcliffe had not books, he had an excellent substitute—brains. His progress at Oxford was simply astounding. In 1675 he took his degree, after which he opened practice in the University town. In 1682 he proceeded M.D., and went out a "Grand Compounder,"—an imposing ceremony in those days, all the members of the college walking in procession, with the candidate himself, to the Convocation House.

In London.

The young doctor, who was rapidly rising into fame, came up to London in 1684 and took a house in Bow Street, then a most fashionable suburb. His reputation had preceded him from Oxford, for his Bow Street practice was soon bringing him in some twenty guineas a day. Some of the doctor's "patients," it is said, even simulated illness in order to enjoy for a few minutes his facetious conversation.

Sir Godfrey Kneller.

Dr. Radcliffe at Bow Street was a neighbour of the distinguished court painter, Sir Godfrey Kneller, who lived in the Piazza, Covent Garden. The painter was a great lover of flowers, and, on a friendship springing up between them, he allowed the physician to have a door made through to his beautiful pleasure-grounds. On one occasion there was a little *contretemps*. Sir Godfrey's servants told their master that the doctor's servants destroyed his flowers. So the painter wrote to the doctor that the said gate must be fastened up. The doctor returned word that Sir Godfrey might do anything he liked save *paint* it. "Did my very good friend, Dr. Radcliffe, say so?" cried Sir Godfrey. "Go you back to him, and after presenting my service to him, tell him that I can take anything from him but his physic!"—a *tu quoque* more biting than true, but one which the aggressor richly merited.

Patronage.

Radcliffe was quite the Abernethy of his day. His independence and his ill-judged levity grew apace with his prosperity. The revolution of 1688 was transpiring, and Radcliffe had to play a politic part, for he had been appointed physician to the Princess Anne. When Dutch William came over to our shores, two of his servants fell sick. Radcliffe attended and cured them, and pocketed a perquisite of five hundred guineas. He was offered the post of physician to the King, but declined, for he did not deem the stability of the throne sufficiently decided. Nevertheless he continued to attend his Majesty, and for the first eleven years of his reign received something like £600 per annum. In 1689 William again required his services. Radcliffe restored his monarch to health in time to take command at the battle of the Boyne.

A "Paying" Patient.

The Princess Anne had married Prince George of Denmark, and out of a progeny of some fourteen in number, only one, William, Duke of Gloucester, an entertaining though precocious child, lived to pass the age of infancy. The little prince having one of his usual fainting fits, the "great doctor" was summoned. He restored the patient, and received from the Queen's Chamberlain an honorarium of a thousand guineas.

Lionised.

Radcliffe now found himself at the very pinnacle of his profession. He was courted by all the great families of the town, and was literally overwhelmed with patients. Indeed, so numerous were the latter, that his neighbour, Dr. Gibbons, was able to pocket a thousand a year from Radcliffe's "overflow" *clientèle*.

A Big Loss.

In the midst of his busy practice Radcliffe was foolish enough to enter with his friend Betterton, the great tragedian, upon a venture in the East Indies. The vessel which was conveying the doctor's and the actor's money was captured by the Marquis de Nesmond. Poor Thomas Betterton was virtually ruined, but the doctor displayed, upon hearing the bad news, a stoical fortitude. He was carousing with some companions at the Bull's Head Tavern in Clare Market when the intelligence was brought. "Never mind," said Radcliffe; "I have no more to do but go up so many pair of stairs to make myself whole again."

A Matrimonial Escape.

About this time the great man began to think of matrimony. He came across the daughter of a wealthy citizen with a marriage portion of £15,000. But the maiden was not for Radcliffe, for a bookkeeper in her father's employ had secured her affections in a way which the following letter from the doctor to the young lady's father will make plain:—"Sir, the honour of being ally'd to so good and wealthy a person as Mr. S——d has push'd me upon a discovery that may be fatal to your quiet, and your daughter's reputation, if not timely prevented. Miss Mary is a very deserving gentlewoman, but you must pardon me if I think her by no means fit to be my wife, since she is another man's already, or ought to be. In a word, it is necessary that she be disposed of to him that has the best claim to her affections. No doubt but you have power enough over her to bring her to confession, which is by no means the part of a physician. As for my part, I shall wish you much joy of a new son-in-law when known, since I am by no means qualified to be so near of kin. Hanging and marrying, I find, go by destiny; and I might have been guilty of the first had I not so narrowly escaped the last. My best services to your daughter, whom I can be of little use to as a physician, and of much less in the quality of a suitor. The daughter of so wealthy a gentleman as Mr. S——d can never want a husband, therefore the sooner you bestow her the better, that the young Hans en Kelder may have the right of inheritance to so large a patrimony. You will excuse me for being so very free with you, for though I cannot have the honour to be your son-in-law, I shall ever take pride in being among the number of your friends, who am,

"Sir, your most obedient servant,

"John Radcliffe."

Radcliffe and Royalty.

In 1694 Queen Mary lay ill from small-pox at Kensington Palace. The court physicians had tried to cure her and had failed, so the Privy Council summoned Radcliffe. Looking at the prescriptions which the court doctors had made for the Queen, Dr. Radcliffe exclaimed, "Her Majesty is a dead woman." Some time afterwards the Princess Anne sent for Radcliffe, who tarried in his attendance. Again the messenger was despatched from St. James's, but Radcliffe declined to hurry himself even for so great a patient as the queen's sister. "Her Highness's distemper," he swore, "was nothing but the vapours. She is in as good a state of health as any woman living, if she could only believe it." Such uncourtly language could not, of course, be tolerated, and the Princess dispensed with his services, and engaged his rival, Dr. Gibbons.

The Doctor's Bill-paying.

If there was anything the doctor hated, it was bill-paying. He was rolling in wealth, yet could not bear to change a sovereign, because, as he said, "it slipped away so quickly." According to his biographer, he frankly admitted his parsimony, "even to spunging." "He could never," says Richardson, "be brought to pay bills without much following and importunity, nor then, if there appeared any chance of wearing them out." A paviour, after long and fruitless attempts, caught him one day just getting out of his chariot. "Why, you rascal," exclaimed the doctor, "do you pretend to be paid for such a piece of work. Why, you have spoiled my pavement, and covered it over with earth to hide your bad work!" "Doctor," said the paviour, "mine is not the only bad work the earth hides." "You dog, you," said the doctor, "are you a wit? You must be poor; come in." And he paid him! Yet Radcliffe, like so many other ornaments of his profession, was one of the most liberal and generous of men.

Mistaken Professions.

The Princess Anne, though she had been much shocked at Radcliffe's rudeness on the occasion above referred to, was forced to send for him again. The little Duke of Gloucester, the heir presumptive to the crown, was very ill, and the doctors who were attending him gave the Princess no hopes of his recovery. So at the eleventh hour Radcliffe was sent for. But it was too late, for the little prince was beyond all human skill. Radcliffe, however, took occasion to rate the court doctors pretty soundly for their ignorant treatment, telling one that it would have been happy for the nation if he had followed his father's occupation as a basket-maker, and the other that he would have done better to have continued making havoc of nouns and pronouns in the quality of a country schoolmaster, rather than to have ventured out of his reach in the practice of an art to which he was a perfect stranger!

An ill-timed Jest.

Though Radcliffe had offended the Princess Anne, he retained the favour of William III, but his Majesty found his blunt language at times rather difficult to swallow. The King, who suffered from dropsy, sent for Radcliffe to prescribe. "Doctor, what do you think of these?" inquired William, showing Radcliffe his swollen ankles. "Why, truly," answered Radcliffe, "I would not have your Majesty's two legs for your three kingdoms." This was the last visit which the King invited Radcliffe to pay him at Kensington. The doctor and his "vapoury" patient died within a few months of each other.

A Thankless World.

In July 1714 Anne was seized with her fatal illness. Radcliffe was again sought out, but, alas, he himself was confined to his house at Carshalton with a bad attack of gout, and could not come. The friends of the Queen reported that Radcliffe *would* not come, and public indignation soon grew loud against him. Radcliffe had recently been elected for the representation of Buckingham, and one of his old friends actually went so far as to move in the House of Commons, four days after the Queen's death, that Radcliffe should be summoned to attend in his place in order to be censured for not waiting on her Majesty in her last extremities. The following extract from a letter to a friend would seem to indicate that Radcliffe had a reason, other than his own illness, for not going. He writes :—"I know the nature of attending crowned heads in their last moments too well to be fond of waiting upon them without being sent for by a proper authority. You have heard of pardons being signed for physicians before a sovereign's demise. However, as ill as I was, I wou'd have went to the queen in a horse litter had either her majesty, or those in commission next her, commanded me so to do." The excitement of the people, however, still continued, and he received several letters threatening him with lynch law if he ever visited London. But he never did. On 1st November 1714 the great doctor followed the Queen to the grave, as Pittis, his biographer, quaintly puts it, "a victim to the ingratitude of a thankless world and the fury of the gout."

